# Detection of *M. tuberculosis* DNA in peripheral blood mononuclear cells of tuberculosis contacts does not associate with blood RNA signatures for incipient tuberculosis

**DOI:** 10.1183/13993003.00479-2024

**Published:** 2024-12-12

**Authors:** Joshua Rosenheim, Markos Abebe, Mulugeta Belay, Begna Tulu, Dawit Tayachew, Metasebia Tegegn, Sidra Younis, David A. Jolliffe, Abraham Aseffa, Gobena Ameni, Stephen T. Reece, Mahdad Noursadeghi, Adrian R. Martineau

**Affiliations:** 1 Division of Infection and Immunity, University College London, London, UK; 2 Armauer Hansen Research Institute, Addis Ababa, Ethiopia; 3 Blizard Institute, Barts and the London School of Medicine and Dentistry, Queen Mary University of London, London, UK; 4 Research Center Borstel, Leibniz Lung Center, Borstel, Germany; 5 Bahir Dar University, Bahir Dar, Ethiopia; 6 National University of Medical Sciences, Rawalpindi, Pakistan; 7 Aklilu Lemma Institute of Pathobiology, Addis Ababa University, Addis Ababa, Ethiopia; 8 Department of Veterinary Medicine, United Arab Emirates University, Al Ain, United Arab Emirates; 9 Infectious Diseases and Vaccines, Kymab, Babraham Research Campus, Cambridge, UK; 10 These authors contributed equally

## Abstract

Human exposure to *Mycobacterium tuberculosis* (Mtb) is thought to result in a spectrum of outcomes, including bacillary clearance, quiescent Mtb infection, incipient tuberculosis (TB), subclinical TB and active TB [1]. Incipient TB – defined as a prolonged asymptomatic phase of early disease preceding clinical presentation as active disease [2] – may be distinguished from quiescent Mtb infection by detection of host gene expression signatures in blood, whose presence associates with increased risk of progression to active TB [3].

## Shareable abstract

**Detection of *M. tuberculosis* DNA in TB contacts’ PBMCs does not associate with blood RNA signatures for incipient tuberculosis** https://bit.ly/4dliODg

*To the Editor:*

Human exposure to *Mycobacterium tuberculosis* (Mtb) is thought to result in a spectrum of outcomes, including bacillary clearance, quiescent Mtb infection, incipient tuberculosis (TB), subclinical TB and active TB [1]. Incipient TB – defined as a prolonged asymptomatic phase of early disease preceding clinical presentation as active disease [2] – may be distinguished from quiescent Mtb infection by detection of host gene expression signatures in blood, whose presence associates with increased risk of progression to active TB [3]. We and others have previously reported detection of Mtb DNA in CD34-positive peripheral blood mononuclear cells (PBMCs) of asymptomatic TB-exposed adults with normal chest radiographs [4–6]. In one such study conducted in Ethiopia [5], administration of isoniazid preventive therapy (IPT) reduced the proportion of HIV-infected individuals in whom this signal was detectable from 95% to 53% (p<0.0001), suggesting that detection of Mtb DNA in PBMCs may represent the presence of viable bacteria. Whether these bacteria provoke a host transcriptional response associated with subclinical disease is not known.

To investigate, we conducted a cross-sectional study with a nested prospective component, as previously described [5]. Briefly, three groups of asymptomatic adults at risk of TB infection (household TB contacts, farm workers with occupational exposure to bovine TB and HIV-infected people attending an outpatient clinic) and living in or around Addis Ababa, Ethiopia, were invited to give a blood sample for processing as described below. Principal exclusion criteria were age <18 years; symptoms of active TB; haemoglobin concentration <10 g·dL^−1^; and presence of any chest radiograph abnormality consistent with active TB. HIV-infected participants who did not have any contraindication to IPT were offered a 6-month course of 300 mg isoniazid and pyridoxine 50 mg daily and invited to return to give a second blood sample for the same laboratory tests following treatment completion. The study was approved by the Ethiopian National Research Ethics Review Committee, Addis Ababa, Ethiopia (ref 310/253/2017). Written informed consent was obtained from all participants. PBMCs were isolated and sorted into CD34-positive and -negative subsets prior to DNA extraction and detection of Mtb DNA, as previously described [5]. For determination of host gene expression signatures, 3 mL blood was collected into Tempus Blood RNA Tubes (Thermo Fisher Scientific) and processed with Tempus Spin RNA Isolation Kits (Thermo Fisher Scientific) to obtain high-quality total RNA. For genome-wide mRNA sequencing, complementary DNA libraries were generated using Kappa HyperPrep kits (Roche) and sequenced on the Illumina NextSeq 550 system using NextSeq 500/550 High Output 75 Cycle Kits (Illumina) RNA sequencing data were mapped to Ensembl Human GRCh38 release 104 using Kallisto (v0.46.1). Scores for eight incipient TB signatures were calculated as reported elsewhere [7–13]. Unpaired t-tests were used to compare gene expression Z-scores of HIV-uninfected participants in whom Mtb DNA was detected *versus* undetected at baseline. In this analysis, a sample size of n=24 per group achieved 92% power to identify a difference in Z-score means of ≥1 (the minimum biologically meaningful difference) with 5% alpha, assuming standard deviation of 1.0 for Z-score values. Paired t-tests were used to compare gene expression Z-scores of HIV-infected participants before *versus* after IPT, with stratification by Mtb PCR responder status. In this analysis, a sample size of n=17 responders achieved 97% power, and a sample size of n=7 non-responders achieved 60% power, to identify a difference in Z-score means of ≥1 with 5% alpha. Between-group differential gene expression was investigated using Deseq2 and false discovery rate (FDR) <0.05.

Gene expression analysis was performed on baseline whole blood samples from randomly selected subsets of 24 out of 33 (72.7%) HIV-uninfected digital PCR (dPCR)-negative participants, 24 out of 89 (30.0%) HIV-uninfected dPCR-positive participants, and 24 out of 67 (35.8%) HIV-infected dPCR-positive participants. Median age of participants contributing data to the current analysis was 35.0 years (interquartile range 25.5 to 42.5 years), and 28 (38.9%) were female. Gene expression analysis was also performed on follow-up blood samples of HIV-infected participants following completion of IPT, of whom 17 (70.8%) were classified as “responders” to IPT (Mtb DNA undetectable at follow-up) and seven (29.2%) were classified as “non-responders” to IPT (Mtb DNA detectable at follow-up).

Cross-sectional analysis of baseline gene expression data from HIV-uninfected participants revealed no differences in Z-scores between Mtb DNA-positive *versus* Mtb DNA-negative participants for any of the eight signatures investigated (figure 1a). Prospective analyses comparing expression of the same eight signatures in HIV-infected participants before *versus* after administration of IPT showed no change in gene expression Z-scores over the course of treatment, either for those in whom Mtb DNA was undetectable at follow-up (“PCR responders”) or for those in whom Mtb DNA was still detected at follow-up (“PCR non-responders”) (figure 1b).

**FIGURE 1:**
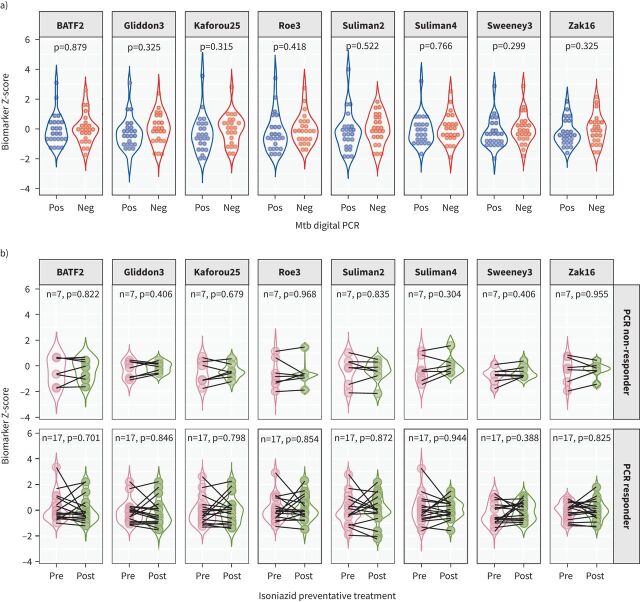
Z-scores for expression of eight different signatures for incipient tuberculosis (TB) in whole blood of asymptomatic adults in Ethiopia. a) Baseline Z-scores by *Mycobacterium tuberculosis* (Mtb) DNA status. b) Z-scores before *versus* after administration of isoniazid preventive therapy among those in whom Mtb DNA was detected at follow-up (“PCR non-responders”, top row) and those in whom Mtb DNA was undetectable at follow-up (“PCR responders”, bottom row). Pos: positive; Neg: negative.

We present findings of the first study to investigate whether detection of Mtb DNA in peripheral blood of asymptomatic adults at risk of *M. tuberculosis* infection associates with blood RNA signatures for incipient TB. No such associations were found on cross-sectional analysis of baseline samples. Moreover, administration of IPT did not influence gene expression signatures in HIV-infected participants in whom Mtb DNA was detected in PBMC at baseline, even though this intervention had previously been shown to reduce the proportion of individuals in whom this signal was detectable after treatment [5].

The lack of association between dPCR-positivity and presence of gene expression signatures for incipient TB in our study suggests that detection of Mtb DNA in PBMC subsets of TB contacts using dPCR does not indicate incipient TB. This interpretation is supported by the observations that no Mtb DNA-positive participants in our study progressed to active TB over 6-month follow-up, and that prevalence of detectable Mtb DNA (79.2%) at baseline was much higher than the risk of progression to active TB (commonly estimated at ∼10%). The high baseline prevalence of Mtb DNA in blood of asymptomatic adults and the persistence of this signal in 53% of participants after offering IPT are both striking findings: further work is ongoing to determine whether detection of Mtb DNA in PBMC associates with the presence of culturable Mtb bacilli and/or Mtb RNA. Our findings chime with those of a linked study in which we report a lack of association between blood RNA signatures and sputum culture status in patients with active TB who have taken 8 weeks of intensive antimicrobial therapy [14]. The analogy is that both studies provide evidence that Mtb may be present in distinct compartments (blood *versus* sputum) without stimulating a detectable “active” host response.

Our study has several strengths. We investigated a comprehensive panel of eight different RNA signatures reported to associate with incipient TB, and found a consistent lack of association with our Mtb DNA signal. Our sample size provided adequate power to detect modest differences in gene expression Z-scores between groups. Inclusion of HIV-infected and -uninfected participants, and contacts of human and animal index cases, improves generalisability of our findings.

Our study also has some limitations. Given the low-income setting, we relied on clinical symptom screening and plain chest radiographs to detect active TB: this approach is less sensitive than positron emission tomography–computed tomography scanning, especially for subclinical or incipient disease. Administration of IPT was not directly observed: given the 6-month duration, it is likely that adherence was suboptimal in at least some participants, which may have contributed to failed clearance of Mtb in some participants.

In conclusion, we report that detection of Mtb DNA in PBMCs of asymptomatic adult TB contacts in Addis Ababa, Ethiopia, does not associate with the presence of host blood gene expression signatures of incipient TB, either at baseline or following completion of preventive therapy. Because we investigated gene expression signatures associated with incipient tuberculosis, rather than incipient disease itself, we cannot determine whether detection of Mtb DNA in PBMC denotes incipient TB. However, given the strong associations between the presence of these signatures and risk of disease progression [3], our findings suggest that detection of Mtb DNA in PBMC subsets of TB contacts using dPCR may be more likely to represent quiescent Mtb infection than incipient or subclinical disease. Ultimately, the prognostic significance of detecting Mtb DNA in PBMC of asymptomatic individuals can only be determined by conducting very large longitudinal studies, powered to determine the positive and negative predictive values of this biomarker for progression to active TB [15].

## Shareable PDF

10.1183/13993003.00479-2024.Shareable1 This PDF extract can be shared freely online.
Shareable PDF ERJ-00479-2024.Shareable

## Data Availability

A de-identified copy of the study database will be made available from the corresponding author (a.martineau@qmul.ac.uk) from the date of publication.
