# Supplementary material for: Detection of M. tuberculosis DNA in peripheral blood mononuclear cells of tuberculosis contacts does not associate with blood RNA signatures for incipient tuberculosis
Source: Eur Respir J. 2024 Dec 12;64(6):2400479. doi: 10.1183/13993003.00479-2024 (PMC11635380; doi:10.1183/13993003.00479-2024)
Supplement: Supplementary file 1 [file ERJ-00479-2024.Shareable.pdf]

EUROPEAN RESPIRATORY JOURNAL
RESEARCH LETTER
J. ROSENHEIM ET AL.

Detection of *M. tuberculosis* DNA in peripheral blood mononuclear cells of tuberculosis contacts does not associate with blood RNA signatures for incipient tuberculosis

Joshua Rosenheim[1], Markos Abebe[2], Mulugeta Belay[2,3], Begna Tulu[4,5], Dawit Tayachew[2], Metasebia Tegegn[2], Sidra Younis[3,6], David A. Jolliffe[3], Abraham Aseffa[2], Gobena Ameni[7,8], Stephen T. Reece[9], Mahdad Noursadeghi[1,10] and Adrian R. Martineau[3,10]

[1]Division of Infection and Immunity, University College London, London, UK. [2]Armauer Hansen Research Institute, Addis Ababa, Ethiopia. [3]Blizard Institute, Barts and the London School of Medicine and Dentistry, Queen Mary University of London, London, UK. [4]Research Center Borstel, Leibniz Lung Center, Borstel, Germany. [5]Bahir Dar University, Bahir Dar, Ethiopia. [6]National University of Medical Sciences, Rawalpindi, Pakistan. [7]Aklilu Lemma Institute of Pathobiology, Addis Ababa University, Addis Ababa, Ethiopia. [8]Department of Veterinary Medicine, United Arab Emirates University, Al Ain, United Arab Emirates. [9]Infectious Diseases and Vaccines, Kymab, Babraham Research Campus, Cambridge, UK. [10]These authors contributed equally.

Corresponding author: Adrian R. Martineau (a.martineau@qmul.ac.uk)

Shareable abstract (@ERSpublications)
Detection of *M. tuberculosis* DNA in TB contacts' PBMCs does not associate with blood RNA signatures for incipient tuberculosis https://bit.ly/4dliODg

Cite this article as: Rosenheim J, Abebe M, Belay M, *et al*. Detection of *M. tuberculosis* DNA in peripheral blood mononuclear cells of tuberculosis contacts does not associate with blood RNA signatures for incipient tuberculosis. *Eur Respir J* 2024; 64: 2400479 [DOI: 10.1183/13993003.00479-2024].

This extracted version can be shared freely online.

Copyright ©The authors 2024.

This version is distributed under the terms of the Creative Commons Attribution Licence 4.0.

Received: 6 March 2024
Accepted: 5 Aug 2024

To the Editor:

Human exposure to *Mycobacterium tuberculosis* (Mtb) is thought to result in a spectrum of outcomes, including bacillary clearance, quiescent Mtb infection, incipient tuberculosis (TB), subclinical TB and active TB [1]. Incipient TB – defined as a prolonged asymptomatic phase of early disease preceding clinical presentation as active disease [2] – may be distinguished from quiescent Mtb infection by detection of host gene expression signatures in blood, whose presence associates with increased risk of progression to active TB [3]. We and others have previously reported detection of Mtb DNA in CD34-positive peripheral blood mononuclear cells (PBMCs) of asymptomatic TB-exposed adults with normal chest radiographs [4–6]. In one such study conducted in Ethiopia [5], administration of isoniazid preventive therapy (IPT) reduced the proportion of HIV-infected individuals in whom this signal was detectable from 95% to 53% (p<0.0001), suggesting that detection of Mtb DNA in PBMCs may represent the presence of viable bacteria. Whether these bacteria provoke a host transcriptional response associated with subclinical disease is not known.
